# Increasing the use of the WHO AWaRe system in antibiotic surveillance and stewardship programmes in low- and middle-income countries

**DOI:** 10.1093/jacamr/dlaf031

**Published:** 2025-03-19

**Authors:** Zikria Saleem, Samia Sheikh, Brian Godman, Abdul Haseeb, Shairyar Afzal, Muhammad Usman Qamar, Mohammad Tarique Imam, Safa S Almarzoky Abuhussain, Mike Sharland

**Affiliations:** Department of Pharmacy Practice, Faculty of Pharmacy, Bahauddin Zakariya University, Multan, Punjab, Pakistan; Department of Pharmacy Practice, Faculty of Pharmacy, Bahauddin Zakariya University, Multan, Punjab, Pakistan; Department of Public Health Pharmacy and Management, School of Pharmacy, Sefako Makgatho Health Sciences University, Ga-Rankuwa 0208, South Africa; Strathclyde Institute of Pharmacy and Biomedical Sciences, University of Strathclyde, Glasgow G4 0RE, UK; Antibiotic Policy Group, Institute for Infection and Immunity, City St George’s, University of London, London SW17 0RE, UK; Clinical Pharmacy Department, Al Rayan National College of Health Sciences and Nursing, Al-Madinah Al-Munawarah, Saudi Arabia; Department of Pharmacy, DHQ Hospital Jhelum, Jhelum, Pakistan; Institute of Microbiology, Faculty of Life Sciences, Government College University Faisalabad, Faisalabad 38000, Pakistan; Department of Clinical Pharmacy, College of Pharmacy, Prince Sattam Bin Abdulaziz University, Al Kharj Pin-11942, Saudi Arabia; Department of Pharmaceutical Practices, College of Pharmacy, Umm Al-Qura University, Makkah 21955, Saudi Arabia; Antibiotic Policy Group, Institute for Infection and Immunity, City St George’s, University of London, London SW17 0RE, UK

## Abstract

**Introduction:**

Antimicrobial resistance (AMR) presents a major global health threat, driven in part by the inappropriate use of antibiotics including in low- and middle-income countries (LMICs). Improving the quality of antibiotic use is a key rationale for the development of the WHO’s AWaRe (Access, Watch and Reserve) system. There is a need to review the uptake of the AWaRe system since its launch to guide future practice.

**Methods:**

A literature search was conducted between 2017, the launch of AWaRe, and 2024. Inclusion criteria were studies that reported on antibiotic use in LMICs using the AWaRe system.

**Results:**

Eighty-five studies were included in the review, of which 56.4% focused on antibiotic use trends, with 28.2% reporting on prescribing patterns; 51.7% of the studies included inpatients. Only 14.1% of studies reported meeting the 2024 United Nations General Assembly (UNGA) AMR recommended target of at least 70% of human antibiotic use being Access antibiotics, with a concerning trend of overuse of Watch antibiotics (68.2% of studies). Dispensing practices revealed significant dispensing of antibiotics without prescriptions especially in Pakistan and Bangladesh. Watch antibiotics were more available but also more expensive than Access antibiotics.

**Conclusions:**

Encouragingly, many LMICs are now reporting antibiotic use via the AWaRe system, including in antimicrobial stewardship programmes (ASPs). Wide variation exists in the proportion of AWaRe antibiotics used across LMICs, with overuse of Watch antibiotics. There is an urgent need for targeted AWaRe-based ASPs in LMICs to meet recent UNGA recommendations. Improving the use, availability and affordability of Access antibiotics is essential to combat AMR.

## Introduction

Antimicrobial resistance (AMR) is associated with considerable morbidity and mortality, estimated at 4.71 million deaths in 2021 and reaching 8.22 million by 2050 if not addressed, accompanied by substantial economic consequences.^1–6^ A key driver of AMR is the overuse of antibiotics.^2,3,7–11^ Following the increasing public health concern regarding AMR and its impact on the achievement of Sustainable Development Goals, particularly target 3.8 by 2030,^12^ the WHO developed the Global Action Plan (GAP) against AMR in 2015.^13^ One of the objectives of the GAP is to optimize the use of antimicrobials.^13,14^ The GAP led to the development of National Action Plans (NAPs) to tackle AMR. There are challenges in both human resources and available finances to fully implement these, especially in low- and middle-income countries (LMICs). There has also been a limited focus so far on national antibiotic policies.^15–18^

In line with fulfilling the GAP, the WHO in 2017 developed the AWaRe classification of antibiotics, based on the WHO Essential Medicines List (EML), in which antibiotics on the EML were grouped as Access, Watch and Reserve (AWaRe).^19^ Antibiotics in the Access group are narrow-spectrum antibiotics, with lower AMR selection pressure and a low toxicity profile, and typically serve as first- and second-line choices for common infections. Antibiotics in the Watch group are broader-spectrum antibiotics, more commonly recommended for use in hospitals, and warrant careful prescribing because of their increased resistance potential The ‘last resort’ Reserve antibiotics are used to treat only serious hospital infections caused by MDR infections, which necessitates their use under strict stewardship protocols.^19–21^ The aim of classifying antibiotics into AWaRe groups is to optimize their empirical use and preserve their future effectiveness, consequently facilitating the achievement of antimicrobial stewardship goals.^20,22^ This is especially important in LMICs where there has been an appreciable increase in the use of antibiotics from the Watch list in recent years resulting in a considerable decrease in the Access-to-Watch Index.^2^ Alongside this, current AMR rates are highest in LMICs, including those in sub-Saharan Africa and Asia.^23,24^ The United Nations General Assembly (UNGA) has recently recommended that at a global level, 70% of all antibiotic use in humans should be Access antibiotics, building on the previous WHO country-level target of 60%.^2,21,25^ As a result, tackling high and inappropriate use of antibiotics from the Watch and Reserve list should be key for any antimicrobial stewardship programmes (ASPs).^2,21,26^ Although ASPs can be more challenging in LMICs due to issues of available personnel and resources,^27^ they are increasingly being undertaken in LMICs with a clear need for context-specific ASPs.^28–33^ This is welcomed as inappropriate use of antibiotics is currently a considerable challenge, particularly in LMICs due to socio-economic, cultural, behavioural, political, regulatory and governance-related factors, which urgently needs addressing to attain UNGA goals.^34–37^

Encouragingly, an increasing number of studies across countries and sectors are also now reporting antibiotic use patterns based on the AWaRe system.^2,9,32,38,39^ In 2022, the WHO launched the AWaRe antibiotic book, which provides detailed guidance on the management of 35 infections for adults and children in both primary care and the hospital sectors.^20,40,41^ However, to date there has not been a comprehensive review evaluating the extent to which the AWaRe system has been adopted across LMICs as a surveillance and stewardship tool. Understanding the challenges and effectiveness of the practical implementation of the AWaRe system and guidance is crucial for developing future strategies to combat AMR.^20^ This includes potentially developing and refining quality indicators based on the AWaRe guidance.^42^ The aim of the study was therefore to assess the uptake of the AWaRe system as part of ASPs in LMIC settings and consider the barriers and enablers of its use. This builds on the studies of Adekoya *et al*.^1^ in 2021 comparing antibiotics included in national essential medicines lists of 138 countries using the AWaRe classification.

## Methodology

A scoping review was conducted to evaluate the publications using the WHO’s AWaRe system since its launch as a tool to assess antibiotic prescribing, dispensing and use in LMICs using the World Bank’s classification system.^43^ The present study is in accordance with the scoping review guidelines.^44^ Objectives, population, study site, and inclusion and exclusion criteria were pre-specified according to the documented methodology.

### Eligibility criteria

This review followed the methodological guidelines (population, concept and context) recommended by the Joanna Briggs Institute for a scoping review to select the inclusion and exclusion criteria.^45^ In accordance with our research question, we defined specific inclusion and exclusion criteria to identify pertinent articles.

#### Inclusion criteria

- English-language publications were included in this analysis.
- Original quantitative research studies were included to enable an assessment of the percentage prescribing of antibiotics in each AWaRe group.
- Studies that focused on countries classified as LMICs as per the World Bank classification system were included in this review.^43^

##### Population

Patients presenting at primary, secondary and tertiary healthcare units whether public or private including community pharmacies and drugstores.

##### Concept

Antibiotic prescribing and dispensing across sectors according to the WHO AWaRe system, rational prescribing as well as antibiotic availability and affordability across sectors and locations.

##### Context

Community and healthcare settings in LMICs.

#### Exclusion criteria

- Case reports, case studies, narrative reviews, discussion articles, systematic reviews, meta-analyses, editorials and conference papers.
- Reviews, expert opinions or commentaries on antibiotic use and ASPs.
- Articles exclusively concentrated on antibiotic resistance microbiology data, susceptibility, antivirals and antifungals.
- Given the scope of this review, non–peer-reviewed studies were excluded.

### Recognizing and generating research questions

This review mainly concentrated on antibiotic use, prescribing, dispensing and sales as well as availability and affordability according to the WHO AWaRe classification among the population of interest in LMICs across healthcare settings. After a detailed initial review of the literature, the following sub-research questions (RQs) were formulated:

- RQ1: What group of AWaRe (Access, Watch, Reserve) antibiotics are most prescribed, dispensed and used?
- RQ2: How are relevant studies evaluating the trend of antibiotic use in LMICs?
- RQ3: What is the uptake of the AWaRe system in LMIC ASPs?

### Search strategy and study selection

Bibliographic databases including Google Scholar, PubMed and Science Direct, were searched systematically by employing relevant keywords and index terms to identify research papers between January 2017 and March 2024. These years were chosen to reflect the launch of the AWaRe classification combined with the start of implementing NAPs to combat AMR.

The keywords utilized in the search strategy followed a combination of three string terms separated by Boolean operators ‘AND’ or ‘OR’. The first-string term included keywords such as ‘AWaRe (Access, Watch, Reserve) Antibiotics, ‘WHO AWaRe group’ and ‘Access, Watch and Reserve’ and ‘Access antibiotics or antimicrobials’ and ‘Watch antibiotics or antimicrobials’ and ‘Reserve antibiotics or antimicrobials’ followed by second ‘antibiotic or antimicrobial prescribing’ and ‘antibiotic or antimicrobial utilization’ and ‘antibiotic or antimicrobials consumption or use’ and ‘antibiotic or antimicrobials dispensing.’ The third string of terms included ‘low- and middle-income countries, low-income countries, less developed countries, and under-developed countries.’ This was complemented by a hand search strategy also pinpointing potentially relevant papers from various journals.

Systematic screening of databases was conducted based on the use of relevant keywords in titles and abstracts by two independent reviewers (S.S) and (S.A) against the inclusion criteria. Duplicate results were removed through Endnote version 20 by S.S. The articles gleaned after the initial screening were then subjected to full-text reading by S.S. The present study also applied forward and backward search strategies to obtain the final number of included articles. Evidence selection warranted consensus between both reviewers (S.S) and (S.A), with disagreements resolved by a third reviewer (Z.S).

### Data extraction

Key attributes of the data including the author(s) name, year of study, country of the study and its income classification, WHO region (Figure S1, available as Supplementary data at *JAC-AMR* Online), duration of study, AWaRe classification version utilized, study site, data source, objective of the study, population, study design, method, and results (Access, Watch, Reserve) and inferences were extracted and recorded on a pre-developed data chart.

The retrieved data were further classified into different themes in line with the objectives of the study: Theme 1—Prescribing patterns of antibiotics in the various LMICs as per WHO AWaRe group (Table 2). Theme 2—Use trends of antibiotics in the various LMICs as per the WHO AWaRe group (Table 3). The utilization patterns were grouped principally based on previous WHO and recent UNGA targets, i.e. ‘< 50%’, ‘50%–60%’, ‘60%–70%’ and ‘>70%’ of total use being Access antibiotics.^2,21,25^ Theme 3—Dispensing practices and sales of antibiotics in the various LMICs as per WHO AWaRe group (Table 4), and Theme 4—Studies reporting availability and affordability of antibiotics in the various LMICs by WHO AWaRe group (Table 5). Given the heterogeneity of studies included in the review, a few papers reported individual antibiotic data (percentages). We classified those antibiotics as per their respective AWaRe class and accumulated the percentages of individual antibiotics in order to calculate the cumulative percentage of each Access, Watch or Reserve class. However, for most papers, extracted data are reported precisely and presented without modification in the review.

The tables have been arranged geographically by WHO region, and subsequently, under each geographical region, arranged in descending chronological order. If there are two or more publications in any one year in any region in any table, the papers are cited in alphabetical order with respect to the first author.

## Results

### Characteristics of included studies

The original search terms identified 85 articles that met the inclusion criteria for final analysis based on their titles and abstracts. Of these 85 studies, 24 (28.2%) focused on prescribing patterns, 48 (56.4%) on use trends, 7 (8.2%) on dispensing and sales, and 6 (7.0%) on the availability and affordability of antibiotics according to the WHO AWaRe system. The characteristics of included studies are described in Table 1.

**Table 1.** Characteristics of included studies (*N* = 85)

Characteristics	No. of studies *n* (%)	References
Patient type		
Outpatients	9 (10.5)	^46–54^
Inpatients	44 (51.7)	^55–93^
Both	10 (11.7)	^94–103^
Not specified	22 (25.8)	^38,104–124^
Gender		
Male	4 (4.7)	^38,50,85,121^
Female	5 (5.8)	^49,59,98,99,113^
Both	47 (55.2)	^46,48,51–58,61,64–79,83,84,86,88,89,91,92,94,95,97,100–102,111,114,118,122,125–127^
Not specified	29 (34.1)	^47,60,62,63,80–82,87,90,93,96,103–110,112,115–117,119,120,123,124,128,129^
Age group		
Neonates	1 (1.1)	^68^
Infants	1 (1.1)	^66^
Children	2 (2.3)	^38,100^
Paediatric	3 (3.5)	^50,94,98^
Adults	11 (12.9)	^47,48,57,59,69,71,83,95,110,111,125^
Geriatric	1 (1.1)	^92^
Mixed	8 (9.4)	^46,56,58,64,65,77,89,126^
All age groups	16 (18.8)	^49,51,52,54,55,67,70,72,76,78,79,84,99,101,102,114^
Not specified	42 (49.4)	^53,60–63,73–75,80–82,85–88,90,91,93,96,97,103–109,112,113,115–124,127–129^
Data source/site		
Primary care hospital	3 (3.5)	^49,57,100^
Secondary care hospital	3 (3.5)	^46,56,128^
Tertiary care hospital	31 (36.4)	^47,50–55,58,59,62,63,65,69,71–73,79,80,83,86,87,89,92–96,101,103,127,129^
Mixed	16 (18.8)	^61,64,67,70,76,78,82,84,85,88,90,97,98,124–126^
Others/not specified/not mentioned	32 (37.6)	^38,48,60,66,68,75,77,81,91,99,102,104–123^
Healthcare sectors		
Public sector	25 (29.4)	^46–51,54,61,65,67,70,74,79,81,83,84,86–89,97,102,103,122,125^
Private sector	5 (5.8)	^66,80,107,118,120^
Both	15 (17.6)	^38,60,75,90,98,99,104,106,110,112–114,116,117,126^
Others/not specified/not mentioned	40 (47.0)	^52,53,55–59,62–64,68,69,71–73,76–78,82,85,91–96,100,101,105,108,109,111,115,119,121,123,124,127–129^
Study type		
Cross-sectional	70 (82.3)	^38,46–48,50–55,57,58,61–64,68,69,71–74,76–81,83,85–88,90–114,116–121,123–125,127–129^
Randomized clinical trial	2 (2.3)	^49,66^
Point prevalence survey	13 (15.2)	^56,59,60,65,67,70,75,82,84,89,115,122,126^
Data classification		
Prescribing pattern	24 (28.2)	^49–53,55–66,98,99,101–103,111,112^
Use trends of antibiotics	48 (56.4)	^46,47,54,67–87,89,91–96,100,104–107,109,113–117,124–129^
Dispensing practices and sales of antibiotics	7 (8.2)	^48,88,108,118,119,122,123^
Availability and affordability of antibiotics	6 (7.0)	^38,90,97,110,120,121^
WHO Regions		
African Region (AFRO)	38 (44.7)	^46,47,51,54,57,59,64,65,69–71,75,77,78,82–84,86,87,95,98–103,105,106,111,112,114,116,117,119,124–126,128^
South-East Asian Region (SEAR)	20 (23.5)	^50,52,55,61,63,66,67,80,81,91–94,96,104,107,108,121,122,129^
Eastern Mediterranean Region (EMR)	20 (23.5)	^48,53,56,58,60,62,68,72–74,79,88–90,109,110,115,120,123,127^
Western Pacific Region	5 (5.8)	^49,85,97,113,118^
Mixed	2 (2.3)	^38,76^
Access antibiotics		
<50%	47 (55.2)	^38,48,50–52,55,56,58–62,64,67,69,70,72–76,78–85,87,89,90,92,94–97,107,108,112,115,118,120,122,123,127,129^
50%–60%	14 (16.4)	^46,47,53,57,63,66,71,77,88,101,104,111,113,126^
60%–70%	12 (14.1)	^54,65,68,99,100,103,105,106,114,119,125,128^
>70%	10 (11.7)	^49,86,93,98,102,110,116,117,121,124^
Data not reported	2 (2.3)	^91,109^

Forty-four (51.7%) studies focused on inpatients,^55–93,106,125–129^ 9 on outpatients (10.5%) and 10 (11.7%) studies included both outpatients and inpatients. However, 22 (25.8%) of the studies did not specify the patient type.^38,104–124^ Both male and female patients were included in 47 (55.2%) studies. Female patients were specifically mentioned in five (5.8%) of the studies,^49,59,98,99,113^ followed by male patients mentioned in four (4.7%).^38,50,85,121^ However, 29 (34.1%) of the studies did not specify the gender of the included patients.

Regarding age group, 16 (18.8%) studies reported all age groups, followed by adults in 11 studies (12.9%). Mixed age, paediatric and adult age groups constituted 8 (9.4%), 3 (3.5%) and 11 (12.9%) studies, respectively. Neonates, infants and geriatrics were included in one study (1.1%) each. However, 42 (49.4%) studies did not specify their age group. Tertiary care hospitals were included as the study sites in 31 (36.4%) studies, followed by a mixed category in 16 (18.8%). Studies conducted in primary and secondary care hospitals were three (3.5%)^49,57,100^ and three (3.5%), respectively.^46,56,128^ The hospital type was not specified in 32 (37.6%) studies. Both the public and private sectors were included in 15 (17.6%) studies. The public sector alone was represented in 25 (29.4%) studies, with the private sector alone represented in 5 (5.8%) studies. However, 40 (47.0%) studies did not specify any sector. Most of the studies were cross-sectional (82.3%) followed by point prevalence surveys in 13 studies (15.2%).^56,59,60,65,67,70,75,82,84,89,115,122,126^ There were only two randomized trials reported (2.3%).^49,66^ The WHO regions included those from the African Region—38 studies (44.7%), South East Asian Region and Eastern Mediterranean Region—20 studies each (23.5%), followed by the Western Pacific Region with 5 studies (5.8%). The remaining two (2.3%) studies were from mixed WHO Regions.

### Characteristics of extracted data and key findings

The design of the current study was such that only quantitative studies were found to be eligible as per the inclusion criterion. Broadly, the extracted data were categorized into antibiotic prescribing patterns, use trends, dispensing practices and sales as well as the availability and affordability of the antibiotics among LMICs according to the WHO AWaRe group. Table 2 summarizes the studies based on the prescribing pattern of antibiotics in LMICs using the WHO AWaRe system, with these data identifying only 28.2% (24/85) of all the studies included in this review. The percentage inclusion of studies in Table 2 by country is as follows: India 20.8% (5/24), Pakistan 16.6% (4/24), Ghana 16.6% (4/24), Zambia 8.3% (2/24) and Tanzania 8.3% (2/24). Countries including Vietnam, Ethiopia, Nigeria, Bangladesh, Democratic Republic of the Congo, Niger, Uganda and those in the Eastern Mediterranean including Iraq, Jordan, Lebanon, Pakistan, Sudan, and Tunisia constituted only 4.1% (1/24) of the total inclusion. These studies examined antibiotic prescription practices and observed high prescribing of Access antibiotics in outpatients. Similarly, among inpatients, high levels of Watch antibiotics were noted, particularly in public tertiary care hospitals.

**Table 2.** Prescribing pattern of antibiotics in low- and middle-income countries by WHO AWaRe groups

Author name/year/ref.	UN classification income	Country	Duration	EML/AWaRe edition	Study site/data source	Objective	Population	Study design/method	Results		
									Access, %	Watch, %	Reserve, %
African Region (AFRO)											
Agyare *et al.*, 2024^65^	LMIC	Ghana	10 to 17 December 2019	2017	Cape Coast Hospital, Ghana	To examine antibiotics prescribing pattern	Inpatients	Point prevalence survey	63.8	36.2	—
Nsojo *et al.*, 2024^103^	LMIC	Tanzania	September 2021 to September 2022	2021	Mbeya Zonal Referral Hospital, Tanzania	To examine prescribing pattern of antibiotics	Inpatients and outpatients	Cross-sectional study	62.4	37.4	0.1
Dereje *et al.*, 2023^111^	LIC	Ethiopia	1–31 October 2022	2019	7 community pharmacies in Dira Dawa city	To examine prescribing indicators and factors by following WHO AWaRe classification	General population	Prospective and cross-sectional study	55.3	43.1	1.7
Ekuma *et al.*, 2023^59^	LMIC	Nigeria	2018–2021 (3 y)	EML = 2021	Tertiary hospital in Uyo, Nigeria	To examine antibiotics prescribed according to AWaRe classification	Inpatients (medical and surgical)	Three-point prevalence survey	48.2	50.5	—
Kakumba *et al.,* 2023^112^	LIC	Democratic Republic of Congo	July–December 2022	2019	District of Tshangu in Kinshasa	To examine antibiotics prescribing according to WHO AWaRe classification	General population	Retrospective and descriptive cross-sectional study	36.5	43.2	-
Mambula *et al.,* 2023^98^	LICs	Niger and Uganda	January to December 2019	EML = 2021 AWaRe = 2021	4 hospitals or health centres in Uganda and Niger	To examine antibiotics prescription in paediatrics at hospital level	Inpatients and outpatients	Retrospective cross-sectional study Mixed-methods study	73.1	26.9	—
Mudenda *et al.,* 2023^101^	LMIC	Zambia	August to September 2022	2021	University teaching hospital in Lusaka, Zambia	To examine antibiotics prescribing pattern in COVID pandemic	Inpatients and outpatients	Cross-sectional study	55.5	43.1	1.4
Amponsah *et al.*, 2022^51^	LMIC	Ghana	2021	2021	University hospital of KNUST (Kwame Nkrumah University of Science and Technology)	To examine prescribing pattern of antibiotics	Outpatients	Cross-sectional study	48.5	47	≤ 3
Hope *et al.*, 2022^64^	LMIC	Ghana	January to December 2021	2019	Bishop Ackon Memorial Christian Eye Centre (BAMCEC), Ghana	To examine antibiotics prescription pattern in acute conjunctivitis	Inpatients	Retrospective cross-sectional study	44	56	—
Mudenda *et al.*, 2022^57^	LIC	Zambia	September to November 2021	2021	5 primary healthcare hospitals in Lusaka	Assessment of antibiotics prescribing pattern	Inpatients	Retrospective cross-sectional study	55	45	—
Darkwah *et al.*, 2021^102^	LMIC	Ghana	December 2019 to March 2020	2019	Police Hospital, Ghana	To examine antibiotics prescribing pattern	Inpatients and outpatients	Descriptive cross-sectional study	74	24	—
Khalfan *et al.,* 2021^99^	LMIC	Tanzania	September 2019	2019	Health facilities in Ilala Municipality, Dar es Salaam	To examine antibiotics prescription pattern	Inpatients and outpatients	Cross-sectional analysis of forms of National Health Insurance Funds insured patients	60.8	33.3	—
South-East Asian Region (SEAR)											
Mandal *et al.*, 2023^50^	LMIC	India	3 mo	2017 EMLc	Tertiary care hospital	To examine prescription rationality	Outpatients	Observational, cross-sectional study	47.3	38.3	0.7
Negi *et al.*, 2023^63^	LMIC	India	July to August 2022 (2 mo)	EML = 2021	Tertiary care institute, north India	To examine antibiotics prescribing pattern	Inpatients	Descriptive cross-sectional study Questionnaire survey was conducted	57.6	38.2	4.1
Priyadharsini *et al.*, 2022^52^	LMIC	India	May to August 2019	2021	Tertiary care teaching hospital, Puducherry	To examine antibiotics prescribing pattern	Outpatients	Prospective cross-sectional study	45	29	Non-specified
Sinha *et al.*, 2022^61^	LMIC	India	Duration not specified	2021	18 public healthcare facilities in 6 districts of Tamil Nadu, India Paediatrics, gynaecology	To examine prescribing pattern of antibiotics according to WHO AWaRe list	Inpatients	Single-point cross-sectional assessment	10.8	89.2	—
Boone *et al.*, 2021^66^	LMIC	Bangladesh	2014–2016	21st list of EML 2018	Hospitalized infants in Dhaka	To examine the prescribing of antibiotics	Inpatients	Birth cohort study, randomized clinical trials	58	38	1
Mugada *et al.*, 2021^55^	LMIC	India	August 2019 to January 2020 (6 mo)	2019	Tertiary care hospital	Utilizing the AWaRe classification to assess the antibiotic prescription trends	Inpatients	Descriptive cross-sectional study	46.8	53.1	—
Eastern Mediterranean Region (EMR)											
Sajjad *et al.*, 2024^53^	LMIC	Pakistan	October to December 2019	2021	Ear nose and throat (ENT) department of Shalamar tertiary care hospital, Lahore	To examine the prescribing pattern of antibiotics	Outpatients	Cross-sectional quantitative analysis	54.9	45	—
Mustafa *et al.*, 2023^58^	LMIC	Pakistan	March to December 2022	2022	4 tertiary care hospitals in Punjab Province	To investigate antibiotics excessive prescribing in COVID-19 patients	Inpatients	Retrospective and medical record-based study	16.7	80.4	2.9
Mustafa *et al.*, 2022^56^	LMIC	Pakistan	October to November 2020 (2 mo)	2018	16 (District Headquarter) DHQ- and (Tehsil Headquarter) THQ-level hospitals in Punjab Province	To examine antibiotics prescribing	Inpatients	Large multicentre research based on PPS (WHO 2018) technique	49.5	45.5	—
Talaat *et al.*, 2022^60^	LMICs	Eastern Mediterranean	October to December 2019	2021	139 hospitals in 7 countries including Tunisia, Pakistan, Jordan, Iraq, Sudan and Lebanon in Eastern Mediterranean Region	To examine antibiotics overuse and overprescription	Inpatients	Point prevalence survey designed from WHO protocol	34.1	64.1	1.8
Musthaq *et al.*, 2018^62^	LMIC	Pakistan	January to June/July 2018	2019	6 medical and surgical wards of teaching hospital of Faisalabad (Independent University Hospital)	To examine antibiotics prescribing and utilization pattern	Inpatients	Retrospective cross-sectional study	25.3	72.9	1.8
Western Pacific Region											
Nguyen *et al.*, 2023^49^	LIC	Vietnam	2019	2021	112 commune health centres (CHCs) in 6 rural districts of Nam Dinh province, northern Vietnam	To examine prescribing of antibiotics in primary care setting	Outpatients	Two cluster-randomized trials Health records viewed retrospectively, registered in Vietnamese Health Insurance Scheme	92.5	5.9	—

EML, Essential Medicines List; EMLc, Essential Medicines List Children; LIC, low-income country; LMIC, low-middle income country; PPS, point prevalence survey.

Table 3 provides a review of the use trends of antibiotics in LMICs by WHO AWaRe group, with relevant data available in 56.4% (48/85) of the total number of studies included in this review. The percentage inclusion of studies in Table 3 by country is as follows: Pakistan, 16.6% (8/48); Sierra Leone, 12.5% (6/48); India, 12.5% (6/48); Ethiopia, 8.3% (4/48); Nigeria and Tanzania, 6.2% (3/48); Ghana and Zambia, 4.1% (2/48); Uganda and Vietnam, 4.1% (2/48). Countries including Myanmar, Sri Lanka, Nepal, Egypt, Bangladesh and Burkina Faso constituted only 2.0% (1/48) of the total inclusion. The findings showed a trend and patterns towards high levels of utilization of antibiotics from the Watch group, with the wide use of ciprofloxacin and third-generation cephalosporins among inpatients of public sector hospitals in Pakistan.^73,89,115^ The current studies noted that inpatients typically receive Watch antibiotics more frequently than Access antibiotics.^96^

**Table 3.** Trends of antibiotic use and study design in low- and middle-income countries as per WHO AWaRe groups

Author name/year/ref.	UN classification income	Country	Duration	EML/AWaRe edition	Study site/data source	Objective	Population	Study design	Results		
									Access, %	Watch, %	Reserve, %
African Region (AFRO)											
Abejew *et al.*, 2024^116^	LIC	Ethiopia	July 2016 to June 2022 (6 y)	2022	Data from the Bahir Dar branch of Ethiopian Pharmaceutical Supply Agency (EPSA) Based on antibiotics distribution data	To examine antibiotics use	General population	Institution based cross-sectional study	77.1	22.9	—
Wieters *et al.*, 2024^77^	LMICs	Sub-Saharan Africa	February 2018 to May 2022	EML = 2021 AWaRe = 2022	12 urban and rural health facilities in Côte d’Ivoire, Burkina Faso, Democratic Republic of Congo, and Republic of South Africa Data from African Network for Improved Diagnostics, Epidemiology and Management of Common Infectious Agents (ANDEMIA)	To examine reported antibiotic use in patients in multicentre ANDEMIA infectious diseases	Inpatients	Descriptive analysis	54.7	44.7	—
Labi *et al.*, 2023^128^	LMIC	Ghana	January 2016 to November 2021 (6 y)	2021	Secondary healthcare sector in Ghana	To examine use of antibiotics pattern	Inpatients	Cross-sectional study WHO methodology for surveillance of antibiotics use	62.7	37.3	—
Lakoh *et al.*, 2023^83^	LIC	Sierra Leone	March to October 2021	2021	Medical wards of 2 hospitals (34 Military Hospital-MH and Makeni Government Hospital-MGH)	To examine antibiotics use and misuse	Inpatients	Cross-sectional study Antibiotics use = DDDs per 100 bed-days	28.2	71.7	—
Lakoh *et al.*, 2023^54^	LIC	Sierra Leone	April 2022	2021	3 tertiary hospitals	To examine antibiotic use and consumption	Outpatients	Descriptive cross-sectional study	69.8	30.2	—
Murungi *et al.*, 2023^106^	LIC	Uganda	2018 to 2021	2023	Data from WHO Global Antimicrobial Resistance and Use Surveillance System, Uganda	To examine national antibiotics use	General population	Cross-sectional study Retrospective data of surveillance of antibiotics use	62.9	14.5	—
Okoro and Lawal, 2023^46^	LMIC	Nigeria	January to December 2021	2021	Public secondary hospital in Nigeria	To examine use and resistance of antibiotics	Outpatients	Cross-sectional retrospective study	51.8	48.2	—
Tirfe *et al.*, 2023^124^	LIC	Ethiopia	2017–2019 (3 y)	2019	Data from Ethiopian Food and Drug Authority (EFDA) import database	To measure antibiotics use	General population	Retrospective cross-sectional study WHO ATC and DDD methodology used	WHO AWaRe classification = 67.8 Ethiopian AWaRe classification = 87.7	WHO AWaRe classification = 32.1 Ethiopian AWaRe classification = 12.2	WHO AWaRe classification = ≤1 Ethiopian AWaRe classification = <1
Dechasa *et al.*, 2022^69^	LIC	Ethiopia	October 2020 to January 2021	EML = 2019 EEML = 2020	Jimma medical centre, tertiary care hospital	To evaluate how frequently patients used antibiotics	Inpatients	Prospective observational research data by semi-structured questionnaire	34	66	—
Lakoh *et al.*, 2022^125^	LIC	Sierra Leone	February to October 2021	EML = 2021 AWaRe = 2021	4 hospitals in 2 different geographical regions of Sierra Leone	To examine antibiotics use in surgical prophylaxis	Inpatients	Prospective cohort study	61.7	38.3	
Kalungia *et al.*, 2022^84^	LIC	Zambia	8–19 November 2021	2021	First- and second-level 10 public hospitals across 10 provinces of Zambia	To examine use of antibiotics in first-and second-level hospitals	Inpatients	Cross-sectional survey conducted using WHO PPS methodology	34.2	65.9	—
Kamara *et al.*, 2022^78^	LIC	Sierra Leone	2020–2021	2019	35 healthcare units in Sierra Leone	To examine antibiotic use in suspected and confirmed COVID-19 patients	Inpatients	Cross-sectional study	33.8	65.8	0.35
Kiggundu *et al.*, 2022^75^	LIC	Uganda	December 2020 to April 2021	2019	13 hospitals	Evaluation of antibiotics utilization through PPS	Inpatients	WHO PPS survey methodology	47.2	44.1	—
Sekoni *et al.,* 2022^95^	LMIC	Nigeria	2017–2019 (3 y)	2021	Lagos University Hospital, Nigeria	To examine antibiotic use	Inpatients and outpatients	Retrospective cross-sectional study	47.5	50.2	2.3
Valia *et al.*, 2022^100^	LIC	Burkina Faso	March 2016 to June 2017	2021	Nanoro Health District, rural hospital	Monitoring antibiotic use and prevalence of antimicrobial resistance	Inpatients and outpatients	Cross-sectional study With acute fever Patient age ≥3 mo visiting hospital	62.5	37.5	—
Aboderin *et al.*, 2021^70^	LMIC	Nigeria	10–27 June 2019	2018	16 government healthcare facilities in Osun state	To assess prevalence of antibiotic use and quality of antimicrobial prescription	Inpatients	Cross-sectional descriptive multicentre survey WHO protocol for PPS on antibiotic use	46.3	53.5	0.2
D’Arcy *et al.*, 2021^126^	LMICs	4 countries (Ghana, Uganda, Zambia and Tanzania)	May to December 2019	AWaRe = 2019	17 hospitals in 4 countries	To examine antibiotics use and prescription pattern	Inpatients	Global PPS (G-PPS) for antibiotics use	57.2	40	—
Do *et al.,* 2021^114^	LMICs	(Mozambique, Ghana, South Africa, Bangladesh, Vietnam and Thailand)	July 2016 to December 2018	2019	Dispensing or purchasing points	To recognize certain interventions to update use of antibiotic	General population	Cross-sectional study	64.6	35	—
Kanu *et al.*, 2021^105^	LIC	Sierra Leone	2017–2019	EML = 2021 AWaRe = 2019	Data from antibiotics imported and registered by Pharmacy Board of Sierra Leone (PBSL)	To examine antibiotics use at national level	General population	Cross-sectional study WHO methodology for surveillance of antimicrobial use	65	31	—
Labi *et al.*, 2021^82^	LMIC	Ghana	September to December 2019	WHO ATC 2013	7 hospitals in Ghana	To examine antimicrobial use	Inpatients	Multicentre observational study Global PPS survey	29.4	32.5	—
Melaku *et al.*, 2021^47^	LIC	Ethiopia	February to March 2019	2019	Jimma Medical Center, Ethiopia	To examine antibiotics consumed among outpatients in hospital	Outpatients	Cross-sectional study	50.6	49.3	—
Amaha *et al.*, 2020^87^	LIC	Eritrea	2014–2018 (5 y)	2019	2 hospitals: Orotta National Referral and Teaching Hospital (ONRTH) and Hazhaz Zonal Referral Hospital (HZRH) in Asmara	To examine the trends of antibiotic use	Inpatients	Retrospective study Antibiotics use = DDD/100 bed-days	18.4	12.0	—
Lakoh *et al.*, 2020^71^	LIC	Sierra Leone	October 2017 to February 2018	2018	Connaught tertiary care hospital	To evaluate adult inpatients’ use of antibiotics	Inpatients	Cross-sectional research	53.7	43.2	—
Mbwasi *et al.*, 2020^117^	LMIC	Tanzania	2017–2019	2017	Public and private sectors	To examine antibiotic use	General population	Cross-sectional study Data analysed according to ATC and DDD	>90	<10	<1
Seni *et al.*, 2020^86^	LMIC	Tanzania	December 2019	2019	6 referral hospitals, United Republic of Tanzania	To examine the use of antibiotics	Inpatients	Analytical cross-sectional study	97.9	1.8	0.3
South-East Asian Region (SEAR)											
Parekh and Dumra, 2024^94^	LMIC	India	January to March 2022	2021	Paediatrics department of tertiary teaching hospital in Ahmedabad, Gujarat	To examine the antibiotics utilization	Inpatients and outpatients	Observational cross-sectional study	33.5	64.6	5.6
Gangopadhyay *et al.*, 2023^129^	LMIC	India	2018–2021 (4 y)	2019	Tertiary care hospital in India	To examine antibiotics utilization in 4 y period in hospital according to AWaRe	Inpatients	Retrospective cross-sectional record-based study	28	60	12.0
Rockwood *et al.*, 2023^93^	LMIC	Sri Lanka	January to December 2019	2021	General surgery ward of tertiary hospital, Sri Lanka	To examine antibiotic use annually	Inpatients	Retrospective descriptive study Antibiotics categorized WHO AWaRe = DDD/100 patient-days	71.3	23.2	5.5
Zirpe *et al.*, 2023^91^	LMIC	India	April 2021 to March 2022	2019	3 ICUs in Puna, India	To examine the impact of ASP on antibiotics use between 2 groups in ICU	Inpatients	Prospective, single-centre study Study divided into 2 parts: Group A or Pre-ASP implementation period Group B or Post-ASP implementation period	—	43.2	56.7
Bansal *et al.*, 2022^96^	LMIC	India	January 2017 to December 2018	2019	Tertiary care hospital, Jaipur, India	To compare over 2 y antibiotics use according to WHO AWaRe	Inpatients and outpatients	Retrospective study WHO GLASS methodology Antibiotics use = DDDs/1000 inhabitant-days	47.2	43.5	5.7
Koya *et al.*, 2022^107^	LMIC	India	2019	2019	Data from PharmaTrac private sector drug sales dataset	To examine systemic antibiotic use	General population	Cross-sectional analysis of antibiotics sales of private sectors	27.0	54.8	1.0
Rashid *et al.*, 2022^67^	LMIC	Bangladesh	February to April 2021	2021	4 government hospitals	To assess antibiotic use at hospital level	Inpatients	A structured survey questionnaire developed based on WHO and Global-PPS design	35.6	64.0	0.1
Baral *et al.*, 2021^80^	LIC	Nepal	2017–2019 (3 y)	2019	Patan tertiary care hospital, Lalitpur District	To examine parenteral antibiotic use annually	Inpatients	Cross-sectional study Antibiotics use = DDD	39.0	55.6	5.35
Pwint *et al.*, 2021^81^	LMIC	Myanmar	2014–2017	Not specified	Public hospitals, Myanmar	To examine trends and patterns of antibiotic use	Inpatients	Cross-sectional study WHO surveillance methodology for antibiotics uses (ATC/DDD classification used)	45	44.7	0.065
Sri Ranganathan *et al.*, 2021^104^	LMIC	Sri Lanka	2017 (retrospectively data extracted in 2018)	EML = 2019	Data from retail pharmacies of private and public hospitals	To examine differences in antimicrobial use between public and private sector	General population	Descriptive cross-sectional study WHO methodology for global programme on surveillance of antimicrobial use	54.1	45.5	—
Senthilkumar *et al.*, 2020^92^	LMIC	South India	November 2018 to April 2019	EML 2019	Rajah Muthiah Medical College and Hospital	To investigate use of antibiotics in geriatrics	Inpatients	Prospective observational study	33.3	54.4	0.7
Eastern Mediterranean Region (EMR)											
Ali *et al.*, 2024^79^	LMIC	Pakistan	January to March 2022 (3 mo)	2017	2 tertiary care hospitals, Islamabad	To examine the pattern of antibiotic use	Inpatients	Descriptive cross-sectional study Antibiotics use = WHO AWaRe	17.6	70	11.6
Jabeen *et al.*, 2023^127^	LMIC	Pakistan	January 2017 to December 2021 (5 y)	2021	Tertiary care hospital, Islamabad	To examine use of antibiotics in tertiary hospital	Inpatients	Descriptive study consisting of retrospective record review of antibiotics dispensed from pharmacy data	12	76	12
Saleem *et al.*, 2023^73^	LMIC	Pakistan	2023	AWaRe book = 2022	Orthopaedic, gynaecological and general surgical wards of Ghurki Trust Teaching Hospital (GTTH), Lahore	Evaluation of surgical wound patients’ antibiotics use	Inpatients	Observational cross-sectional study	43.0	56.9	—
Hussein *et al.*, 2022^72^	LMIC	Egypt	2019–2020	2020	Beni-suef University Hospital (BUH)	Evaluation of changes and antibiotics use before and after COVID-19	Inpatients	Descriptive analysis Antibiotics use = DDD/100 occupied bed-days (OBD) (%)	23	75.4	1.5
Khalid *et al.*, 2022^68^	LMIC	Pakistan	January to December 2020 (1 y)	2019	Paediatric medicine department of Nishtar University Hospital, Multan	Rational antibiotics use in neonates	Inpatients	Descriptive cross-sectional chart review study	65.5	32.7	—
Mustafa *et al.*, 2022^89^	LMIC	Pakistan	December 2021 to January 2022	2021	3 tertiary care children’s hospitals in Punjab Province	Examined use of antibiotics	Inpatients	Multicentre PPS according to WHO methodology	21.6	76.6	1.7
Saleem *et al.*, 2021^115^	LMIC	Pakistan	October to December 2017	2021	5 community pharmacies, Lahore	Evaluation of antibiotics use rate	General population	Multicentre repeated prevalence survey	41.0	52.0	0.2
Ul Mustafa *et al.*, 2021^74^	LMIC	Pakistan	August to September 2020	2019	5 district hospitals in Punjab Province	Evaluation of antibiotics use in COVID-19 patients	Inpatients	Retrospective cohort study Antibiotics use = DDD/100 OBD	7.5	82.5	—
Malik and Figueras, 2019^109^	LMIC	Pakistan	April 2014 to March 2019 (5 y)	EML = 2019	Data from IQVIA Pakistan data (retail) on antibiotics sales	Evaluation of cephalosporin and fluoroquinolones antibiotics utilization	General population	Retrospective study ATC/DDD methodology Antibiotics use = DIDs (DDDs/1000 inhabitant/day)	Not-specified	61.5	Not-specified
Western Pacific Region											
Dat *et al.*, 2022^85^	LIC	Vietnam	March to July 2019	2019	In 5 provinces, 51 critical care units in primary and secondary hospitals, Vietnam	To examine empirical antibiotic use in critical care units (CCUs)	Inpatients	Cross-sectional multicentre study	24.1	87.3	0.54
Nguyen *et al.*, 2020^113^	LIC	Vietnam	September 2017 to July 2018 (1 y)	2019	15 private pharmacies; 5 public health pharmacies	Reviewing community-level antibiotic use	General population	Cross-sectional research ABACUS study (Antibiotic Access and Use)	50.7	47.3	—
All regions except the European and American Regions											
Ingelbeen *et al.*, 2021^76^	LMICs	4 countries	January 2013 to October 2014	EML 2019	6 healthcare facilities in 4 LMICs (Cambodia, Democratic Republic of Congo, Nepal and Sudan)	To examine antibiotic use for persistent fever, prior to seeking medical care	Inpatients	The NIDIAG-Fever (Neglected Infectious disease Diagnosis-Fever) cross-sectional study	34.5	64.4	—

ASP, antimicrobial stewardship programme; ATC, Anatomical Therapeutic Chemical Classification; DDD, defined daily dose; DIDs, DDDs per 1000 inhabitants per day; EEML, Ethiopian Essential Medicines List; GLASS, Global Antimicrobial Resistance and Use Surveillance System; G-PPS, global point prevalence survey; IQVIA, I (IMS Health), Q (Quintiles), and VIA (by way of); LIC, low income country; LMIC, low-middle income country; OBD, occupied bed days; PPS, point prevalence survey.

Table 4 summarizes the dispensing practices and sales of antibiotics according to WHO AWaRe group, accounting for 8.2% (7/85) of the total studies included in this review. The included studies that collated sales data collated such data predominantly from pharmacies and retail drugstores. The percentage inclusion of studies in Table 4 as per country is as follows: most studies came from Pakistan 28.5% (2/7), with the other countries, including India, Bangladesh, Ghana, Syria and Vietnam, constituting only 14.2% each (1/7) of total inclusion. The review noted that in Pakistan, pharmacies and medical stores typically sell antibiotics without the legal order of prescriptions,^123^ potentially exacerbated by concerns with the classification of antibiotics in the current drug laws.^130^ In Pakistan self-purchasing is increasing, especially the purchasing of Watch antibiotics, which is higher among healthcare workers from the public health facilities sectors.^88^ In general, antibiotics were widely prescribed among outpatients, with a significant proportion of antibiotics dispensed inappropriately.

**Table 4.** Dispensing practices and sales of antibiotics in low- and middle-income countries by WHO AWaRe groups

Author name/year/ref.	UN classification income	Country	Duration	EML/AWaRe edition	Study site/data source	Objective	Population	Study design/method	Results		
									Access, %	Watch, %	Reserve, %
African Region (AFRO)											
Ngyedu *et al.*, 2023^119^	LMIC	Ghana	July 2021 and January to March 2022	2021	4 cities (Cape Coast, Accra, Tamale, Kumasi) in Ghana	To examine without-prescription antibiotics selling among pharmacies and drug sellers	General population (community-level patients)	Prospective cross-sectional study using simulated client methodology Antibiotics offered for 2 conditions (urinary tract infection and paediatric diarrhoea)	63.0	37	—
South-East Asian Region (SEAR)											
Islam *et al.*, 2022^122^	LMIC	Bangladesh	January to July 2021	2021	128 pharmacies	Assess pattern of antibiotic dispensing	General population	Study was conducted as a component of a national PPS	36.4	53.6	10.0
Mehta *et al.*, 2022^108^	LMIC	India	2020	2019	Sales market in India	To examine systemic antibiotics sales according to WHO	General population	Descriptive cross-sectional study on market sale of antibiotics in 2020	47.9	46.7	1.0
Eastern Mediterranean Region (EMR)											
Mustafa *et al.*, 2023^88^	LMIC	Pakistan	May to August 2022	AWaRe = 2021	Public sectors in 6 districts, Punjab Province	To evaluate purchasing, self-medication practices of antibiotics among professionals	Inpatients	Descriptive cross-sectional study	56.5	86.5	—
Aljadeeah *et al.*, 2020^48^	LIC	Syria	June 2018 to May 2019 (12 mo)	2019	Health insurance data from Syrian government	To examine dispensing pattern of antibiotics	Outpatients	Cross-sectional study	32.5	65.8	0.8
Saleem *et al.*, 2020^123^	LMIC	Pakistan	2020	2017	Pharmacies and medical stores	Antibiotic sales without a prescription	Community-level outpatients	Cross-sectional multicentre study Simulated client technique used	31.7	49.3	19.0
Western Pacific Region											
Nguyen *et al.*, 2022^118^	LIC	Vietnam	2 y period	2019	360 private drugstores in 9 provinces in Vietnam	To examine antibiotics dispensing and knowledge about antibiotics	General population	National cross-sectional survey Study questionnaires and face-to-face interviews	40.6	59.3	—

EML, Essential Medicines List; LIC, low-income country; LMIC, low-middle income country, PPS, point prevalence survey.

Table 5 summarizes the findings of multiple studies examining the availability and affordability of antibiotics in diverse healthcare settings. This domain included 7.0% (6/85) of the included studies. In this domain, 33.3% (2/6) of the studies were conducted in Pakistan. The review findings suggest that the overall availability of Watch antibiotics is greater in healthcare settings in Pakistan, resulting in their high use. However, despite being more affordable, Access antibiotics were typically less available than Watch antibiotics in Pakistan. This may be because Watch antibiotics are typically higher priced, with a greater potential for profit.

**Table 5.** Studies reporting availability and affordability of antibiotics in low- and middle-income countries by WHO AWaRe group

Author name/year/ref.	UN classification income	Country	Duration	EML/AWaRe edition	Study site/data source	Objective	Population	Study design/method	Results		
									Access, %	Watch, %	Reserve, %
South-East Asian Region (SEAR)											
Gautham *et al.*, 2022^121^	LMIC	India	February to May 2017	2019	2 districts (Birbhum, South24Parganas) in state of Bengal	To examine availability, affordability and prices of antibiotics stocked by informal providers (IPs)	General population	Cross-sectional survey Provider questionnaire and antibiotics stock sheet are the component of survey tool	71	84	—
Eastern Mediterranean Region (EMR)											
Darwish Elhajji *et al.*, 2023^110^	LMIC	Jordan	2023	2023	Data from 4 sources: (i) WHO AWaRe classification, 2023; (ii) 23rd EML list, 2023; (iii) ATC/DDD index; (iv) Jordan Food and Drug Administration (JFDA)	To examine registered antibiotics according to AWaRe and cost, availability and affordability	General population	Cross-sectional study	100.0	72.3	—
Rafi *et al.,* 2024^90^	LMIC	Pakistan	August to September 2020	EML = 2021 AWaRe = 2021	5 public tertiary care hospitals, Lahore	To examine the availability and comparison of essential medicines according to AWaRe classification	Inpatients	Cross-sectional study conducted according to WHO/HAI methodology	37.6	64.8	43.3
Saleem *et al.*, 2021^120^	LMIC	Pakistan	February to March 2018	EML = 2017	16 private pharmacies in 4 different regions of Lahore	To examine WHO key access antibiotics price, availability and affordability	General population	Cross-sectional survey WHO/HAI methodology: measuring prices, availability of originator brand (OB) and low-price generic (LPG), and affordability	25.1	56.7	—
Western Pacific Region											
Dat *et al.*, 2020^97^	LIC	Vietnam	2018	2019	52 provincial health authorities and 30 public hospitals in Vietnam Regulatory authority	To examine availability, use and price of antibiotics	Inpatients and outpatients	Cross-sectional study Antibiotics classification = AWaRe Antibiotics use and pricing = DDDs	47.2	52.4	0.1
All regions except the European and American regions											
Knowles *et al.*, 2020^38^	LMICs	20 countries	1997 to 2000	EMLc =2017	Public, private and charity-based healthcare centres	Evaluation of availability and use of antibiotics	General population	Cross-sectional health survey	43.5	31.4	—

ATC, Anatomical Therapeutic Chemical Classification; DDD, daily defined dose; EML, Essential Medicines List; EMLc, Essential Medicines List Children; HAI, hospital acquired infection; LIC, low-income country; LMIC, low-middle income country.

### Use of AWaRe antibiotics

We subsequently summarized the data on the patterns of use of Access antibiotics from the 85 studies as shown in Table 1. We identified studies where a range of Access antibiotic use could be explored. As stated, we empirically divided the data into the following ranges: ‘<50%’, ‘50%–60%’, ‘60%–70%’ and ‘>70%’ of total use being Access antibiotics. Our findings suggest overall patterns of high Watch antibiotic use identified in most reported ASPs contrast with the recent UNGA global recommendation of 70% Access group antibiotics. Our findings note that only 11.7% of studies reported >70% antibiotic use from the Access group antibiotics. In 55.2% of the studies, the overall utilization of Access antibiotics was <50%, with the Watch group as high as 68.2% reported in the studies (Table S1).

## Discussion

To our knowledge, this scoping review is the first attempt to summarize the studies in LMICs that have reported patterns of antibiotic use and stewardship by the AWaRe system since its launch in 2017. Encouragingly, the results reported here show the rapid uptake of the use of the AWaRe system as the key method of analysis and reporting of ASP study outcomes. The review included studies from a wide range of healthcare institutions, across primary, secondary and tertiary care in LMICs, with both inpatient and outpatient populations as well as community settings including pharmacies. The data still predominantly cover tertiary care settings, with only a few studies reporting antibiotic use at a primary care level,^49,57,100^ and secondary care hospitals.^46,128^ The study findings showed significant variation in the relative use of the Access, Watch and Reserve groups of antibiotics across the different regions and countries, similar to other studies,^9,131^ with particularly high use of Watch antibiotics among the different sectors across the LMICs, comparable with other worldwide studies.^2,132^

A recent study by Klein *et al*.^2^ noted high consumption of Watch antibiotics in LMICs, with the Access-to-Watch Index declining from 0.96 in 2016 to 0.92 in 2023, which is a concern.^133^ Likewise, Pauwels *et al.*^132^ noted similar findings from a hospital point prevalence survey of 69 countries. Among our study findings, the wide range of Watch antibiotics in the market is noteworthy, possibly influencing their utilization, as illustrated in India where Sulis *et al.*^134^ noted that 47.6% of the total antibiotic prescriptions constituted Watch antibiotics. There was a similar situation in Bangladesh.^122^ A cross-sectional study by Rafi *et al*.^90^ in Lahore, Pakistan, also reported limited use and availability of Access antibiotics alongside considerable availability of Watch antibiotics at the surveyed sites. The pharmaceutical markets of India and Pakistan influence the prescriber’s selection of antibiotics, as there are typically more branded generics of Watch antibiotics available than Access antibiotics, typically at higher prices.^109,135,136^ We will be exploring this further in future studies especially as the availability of Access antibiotics has also been seen as major problem in other LMICs.^38^ As a result, there are currently clear difficulties for some LMICs in following the WHO’s recommendation of maintaining adequate availability, as well as use, of Access antibiotics,^21,137^ with challenges and issues including the quality of available antibiotics, the types of formulations available, and their prices. The latter is a particular issue in LMICs, where there are currently high co-payment issues and issues of affordability. We will also be following this up in future studies. The prescriber’s perception of AMR,^138,139^ and the high rates of AMR,^10,140–142^ are also important factors influencing the use of broad-spectrum Watch antibiotics in LMICs. Another notable reason for the high use of Watch antibiotics in LMICs is the lack of diagnostic capabilities, exacerbating empirical prescribing with broad-spectrum antibiotics.^27^ This urgently needs to be addressed going forward.

Our analysis of the data also encouragingly demonstrated high use of Access antibiotics in outpatient settings in a number of LMICs. A narrative review by Chigome *et al.*^143^ reported similar findings from South Africa. In contrast, Robertson *et al.*^144^ studied antimicrobial use in different European countries, and only 14 of 30 European Union/European Economic Area (EU/EAA) countries achieved the WHO target of 60% utilization of Access category antibiotics consistently over a 5 year period. Simmons *et al.*^145^ studied antimicrobial sales in eight high-income countries (HICs), and the median percentage sale of Access category antibiotics was reported to be 68.3% in 2018. Encouragingly, the overall sale of Access antibiotics improved during the study period (2013–2018). The disparity between LMICs and HICs is very prominent; weakened regulatory capacity and a comparative lack of ASPs are the major impediments in achieving the WHO target, exacerbated by resource and personnel issues.^27,146^ However, this is starting to change.^30,39,147–149^

Our study noted that a number of LMICs, including Bangladesh, Pakistan and Ghana, struggle to curtail non-prescription antibiotic sales, with a high use of self-medication with antibiotics.^88,119,130^ Two different simulated client studies from Pakistan and Indonesia highlighted similar concerns, with antibiotics being dispensed without a prescription in 96.9% and 69% of mystery shoppers’ visits, respectively.^123,150^ Previous studies have also demonstrated that many LMICs have weak regulatory enforcement mechanisms to reduce inappropriate sales of antibiotics without a prescription.^151^ Many social, cultural, behavioural and economic circumstances contribute to this problem,^35,152^ which also includes patient requests for antibiotics exacerbated by their limited knowledge of antibiotics and AMR.^153–157^ We will also be following this up in future studies, especially as the use of antibiotics in ambulatory care in LMICs can account for 90% or more of total antibiotic use, with non-prescription antibiotic sales often accounting for an appreciable proportion of this.^152,158^ Many included studies (55.3%) did not report data on the use of Reserve antibiotics, highlighting their limited use, as shown in Table S1. This is comparable to other studies that also noted appropriately very low use of this category of WHO’s AWaRe system.^9,132^ However, individual country-level statistics for this group of antibiotics may differ from our broader context analysis.

We are aware of a number of limitations with this review. The included studies used diverse methodologies including cross-sectional surveys and retrospective reviews, which complicates direct comparison and limits generalizability across countries and settings. The reliance on cross-sectional data further limits the ability to assess the long-term impact of any stewardship intervention, and the absence of qualitative insights reduces understanding of behavioural and systematic factors. The available data on antibiotic use among primary and secondary care hospitals were limited, and two studies did not report on the use of Access antibiotics.^91,109^ Most studies also originated from capitals and districts, which will not be a comprehensive representation of the entire country. This review also included multiple studies reporting heterogeneous data of different patient types, demographics and methodologies. Lastly, potential publication bias and inconsistent data collection standards may skew the findings, highlighting the need for more standardized, longitudinal and diverse research on AWaRe-based antibiotic stewardship in LMICs. Nonetheless, we believe this review provides a valuable first comprehensive overview of antibiotic use in LMICs through the WHO AWaRe lens, synthesizing data on prescribing patterns, consumption trends and stewardship practices. By consolidating findings across diverse regions and settings, it offers critical insights into antibiotic stewardship needs and highlights gaps for targeted intervention in resource-limited contexts. We believe several recommendations to help facilitate the future integration of the AWaRe system into LMIC ASPs can be made from this review (Box 1). Overall, this study confirms the rapid uptake of the AWaRe system, reflecting that the WHO AWaRe system is now the recognized method for antibiotic surveillance and stewardship globally. The upcoming review of the Global Action Plan can assist with the further development of the AWaRe system as a major policy tool to improve the quality of global antibiotic use alongside the development of pertinent indicators.^13,14^

**Recommendations**

- Listing of antibiotics by the WHO AWaRe group on the National Essential Medicine List of all LMICs to help achieve their NAP goals.
- Government or private healthcare entities should integrate the WHO’s AWaRe system into their formularies, and routinely monitor and report antibiotic use by AWaRe groupings.
- The WHO’s AWaRe system should be integrated into all national standard treatment guidelines (STGs). The WHO’s AWaRe book offers comprehensive guidance regarding the treatment protocol for over 30 infections occurring in primary and secondary care settings and can act as a template to inform national and local STGs.
- Future ASP studies should use the WHO AWaRe system as a measure to assess and report antimicrobial stewardship programme (ASP) interventions. Adherence to AWaRe book recommendations should help reduce inappropriate use of Watch antibiotics, particularly in primary care settings.^11^
- AWaRe system-based quality indicators and quantity metrics should be introduced into local and national ASPs in primary, secondary and tertiary care, building on current initiatives.^42^
- Governments should establish stricter policies to control self-medication, especially inappropriate dispensing of antibiotics without prescriptions including for patients with self-limiting conditions, particularly those in the Watch and Reserve categories.
- The AWaRe system acts as a simple tool to monitor antibiotic use surveillance. Adopting the AWaRe system as the standardized method to collect data on antibiotic utilization across all sectors within countries, and subsequent classification of antibiotics by AWaRe groups, should be included in all future surveillance of antibiotic use at local, regional and national levels.

### Conclusion

This study has demonstrated that the WHO AWaRe system has had rapid uptake globally as the standard method to report patterns of antibiotic use in LMICs. Box 1 summarizes our recommendations of how the AWaRe system could be more closely integrated into future ASPs in LMICs. Strengthening ASPs with the full integration of the AWaRe system into national formularies and treatment guidelines could also assist with the standardization of surveillance of antibiotic use measuring outcomes of future ASP and antibiotic policy interventions, committed to by the 2024 UNGA-AMR recommendations.

## Supplementary Material

dlaf031_Supplementary_Data

## Supplementary data

Figure S1 and Table S1 are available as Supplementary data at *JAC-AMR* Online.
