# Supplementary material for: Increasing the use of the WHO AWaRe system in antibiotic surveillance and stewardship programmes in low- and middle-income countries
Source: JAC Antimicrob Resist. 2025 Mar 19;7(2):dlaf031. doi: 10.1093/jacamr/dlaf031 (PMC11919820; doi:10.1093/jacamr/dlaf031)
Supplement: dlaf031_Supplementary_Data [file dlaf031_supplementary_data.pdf]

Supplementary Data

Figure S1 - Geographical regions where the included studies were conducted.

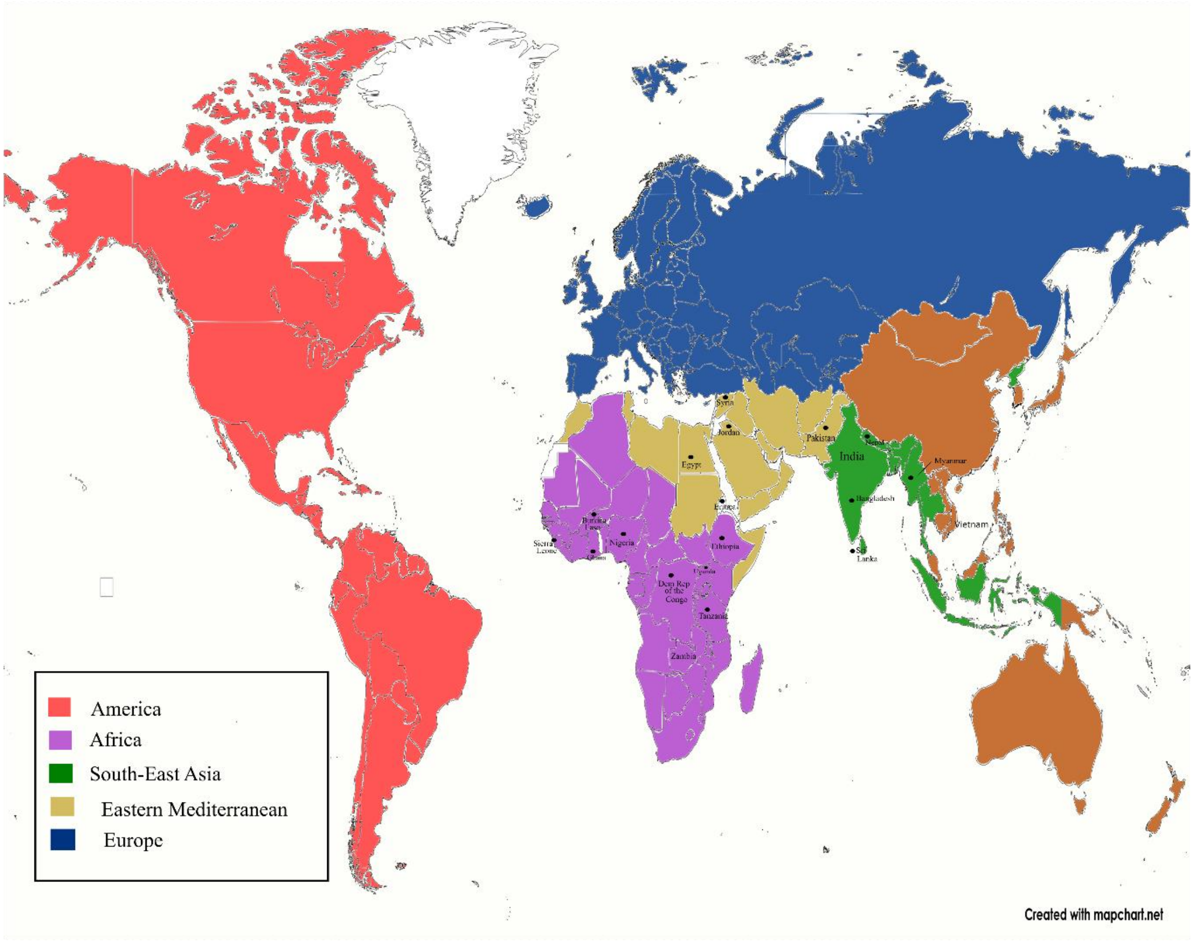

NB: Colors refer to different WHO Regions of the World

Table S1: Percentage Use of AWaRe group antibiotics in studies reporting ASPs in LMIC.

	N (%)	References
Access group		
<50%	47 (55.2)	[1-47]
50-60%	14 (16.4)	[48-61]
>60%	22 (25.8)	[62-83]
Data not reported	2 (2.3)	[84, 85]
Watch group		
≤40%	27 (31.7)	[17, 42, 46, 49, 51, 56, 62, 63, 65, 67-69, 71-77, 79, 81, 86-90]
>40%	58 (68.2)	[1-12, 14-16, 18-20, 22-37, 39-41, 43, 45, 47, 48, 50, 53-55, 57-61, 82-85, 91-94]
Reserve group		
≤10%	32 (37.6)	[2, 4, 6, 8, 10, 11, 19, 24, 25, 27, 29-34, 37, 39-41, 45, 49-51, 58, 77, 79, 81, 86, 87, 90, 92, 93]
>10%	6 (7.0)	[24, 26, 28, 35, 43, 84]
Data not reported	47 (55.2)	[1-3, 5, 7, 9, 12, 14, 15, 17, 18, 20, 22, 23, 36, 42, 46-48, 53-57, 59-63, 65, 67-69, 71-76, 78, 82, 83, 85, 88, 89, 91, 94]

NB: The range or the threshold values under each AWaRe category (Access, Watch and Reserve) are adopted from literature and the online portal of WHO AWaRe [8, 95-101]

References

1. Mugada, V., et al., *Evaluation of prescribing patterns of antibiotics using selected indicators for antimicrobial use in hospitals and the access, watch, reserve (AWaRe) classification by the World Health Organization.* Turkish Journal of Pharmaceutical Sciences, 2021. **18**(3): p. 282.
2. Mustafa, Z.U., et al., *Antibiotic consumption among hospitalized neonates and children in Punjab province, Pakistan.* Expert review of anti-infective therapy, 2022. **20**(6): p. 931-939.
3. Kakumba, J.M., et al., *Evaluation of Antibiotic Prescribing Pattern Using WHO Access, Watch and Reserve Classification in Kinshasa, Democratic Republic of Congo.* Antibiotics, 2023. **12**(8): p. 1239.
4. Mustafa, Z.U., et al., *Antibiotic overprescribing among neonates and children hospitalized with COVID-19 in Pakistan and the implications.* Antibiotics, 2023. **12**(4): p. 646.
5. Ekuma, A., et al., *AWaRe classification of antibiotics prescribed within 2018-2021 for hospitalised medical and surgical patients in Uyo, Nigeria.* The Pan African Medical Journal, 2023. **46**.
6. Talaat, M., et al., *Over-prescription and overuse of antimicrobials in the Eastern Mediterranean Region: the urgent need for antimicrobial stewardship programs with access, watch, and reserve adoption.* Antibiotics, 2022. **11**(12): p. 1773.
7. Sinha, I., et al., *Access–Watch ratio based on access, watch, and reserve classification of antibiotics in public health facilities of Tamil Nadu.* Indian Journal of Public Health, 2022. **66**(3): p. 352-354.

8. Mushtaq, S., et al., *Assessment of antibiotic prescription pattern using who prescribing indicators and aware categorization of antibiotics.* Methodology, 2018. **15**(11): p. 2872-2875.
9. Hope, P.K.F., et al., *Appropriateness of Antibiotic Prescribing for Acute Conjunctivitis: A Cross-Sectional Study at a Specialist Eye Hospital in Ghana, 2021.* International Journal of Environmental Research and Public Health, 2022. **19**(18): p. 11723.
10. Rashid, M.M., et al., *Pattern of antibiotic use among hospitalized patients according to WHO access, watch, reserve (AWaRe) classification: findings from a point prevalence survey in Bangladesh.* Antibiotics, 2022. **11**(6): p. 810.
11. Aboderin, A.O., et al., *Antimicrobial use among hospitalized patients: A multi-center, point prevalence survey across public healthcare facilities, Osun State, Nigeria.* Germs, 2021. **11**(4): p. 523.
12. Dechasa, M., et al., *Antibiotics use evaluation among hospitalized adult patients at Jimma Medical Center, southwestern Ethiopia: the way to pave for antimicrobial stewardship.* Journal of Pharmaceutical Policy and Practice, 2022. **15**(1): p. 84.
13. Hussein, R.R., et al., *Antibiotic consumption in hospitals during COVID-19 pandemic: a comparative study.* Journal of Infection in Developing Countries, 2022. **16**(11): p. 1679-1686.
14. Saleem, Z., et al., *Antibiotic utilization patterns for different wound types among surgical patients: findings and implications.* Antibiotics, 2023. **12**(4): p. 678.
15. Ul Mustafa, Z., et al., *Antimicrobial consumption among hospitalized patients with COVID-19 in Pakistan.* SN comprehensive clinical medicine, 2021. **3**(8): p. 1691-1695.
16. Mustafa, Z.U., et al., *Antimicrobial utilization among neonates and children: a multicenter point prevalence study from leading children's hospitals in Punjab, Pakistan.* Antibiotics, 2022. **11**(8): p. 1056.
17. Labi, A.-K., et al., *Antimicrobial use in hospitalized patients: a multicentre point prevalence survey across seven hospitals in Ghana.* JAC-Antimicrobial Resistance, 2021. **3**(3): p. dlab087.
18. Kiggundu, R., et al., *Point prevalence survey of antibiotic use across 13 hospitals in Uganda.* Antibiotics, 2022. **11**(2): p. 199.
19. Senthilkumar, S., et al., *Study on antibiotic use among geriatric patients based on anatomical therapeutic classification or defined daily dose methodology and world health organization-essential medicine list access, watch and reserve concept in tertiary care hospital of South India.* Int J Basic Clin Pharmacol, 2020. **9**(07): p. 1106-1113.
20. Ingelbeen, B., et al., *Antibiotic use prior to seeking medical care in patients with persistent fever: a cross-sectional study in four low-and middle-income countries.* Clinical Microbiology and Infection, 2021. **27**(9): p. 1293-1300.
21. Bansal, A., R. Sharma, and R. Prakash, *Adoption of the World Health Organization access, watch reserve index to evaluate and monitor the use of antibiotics at a tertiary care hospital in India.* Perspectives in Clinical Research, 2022. **13**(2): p. 90.
22. Lakoh, S., et al., *Antibiotic use and consumption among medical patients of two hospitals in Sierra Leone: a descriptive report.* BMC Infectious Diseases, 2023. **23**(1): p. 737.
23. Kalungia, A.C., et al., *Antibiotic use and stewardship indicators in the first-and second-level hospitals in Zambia: findings and implications for the future.* Antibiotics, 2022. **11**(11): p. 1626.
24. Jabeen, N., et al., *Estimating antibiotics consumption in a tertiary care hospital in Islamabad using a WHO's defined daily dose methodology.* Antimicrobial Resistance & Infection Control, 2023. **12**(1): p. 132.

25. Dat, V.Q., et al., *Antibiotic use for empirical therapy in the critical care units in primary and secondary hospitals in Vietnam: a multicenter cross-sectional study.* The Lancet Regional Health–Western Pacific, 2022. **18**.
26. Gangopadhyay, T., et al., *Antibiotic Consumption in a Tertiary Care Hospital during the Pre-COVID-19 and COVID-19 Years: A Pharmacy-based, Retrospective Study.* Hamdan Medical Journal, 2023. **16**(3): p. 263-270.
27. Kamara, I.F., et al., *Antibiotic use in suspected and confirmed COVID-19 patients admitted to health facilities in Sierra Leone in 2020–2021: practice does not follow policy.* International journal of environmental research and public health, 2022. **19**(7): p. 4005.
28. Ali, N., et al., *Use of Antibiotics in Tertiary Care Hospitals of Meteropolitan City Islamabad.* Journal of Health and Rehabilitation Research, 2024. **4**(1): p. 1163-1169.
29. Baral, P., et al., *Annual consumption of parenteral antibiotics in a tertiary hospital of Nepal, 2017–2019: A cross-sectional study.* Public Health Action, 2021. **11**(1): p. 52-57.
30. Parekh, R.K. and G.H. Dumra, *A cross sectional observational study to evaluate utilization of antimicrobials in paediatric department of a tertiary care teaching hospital.* International Journal of Basic & Clinical Pharmacology, 2024. **13**(1): p. 95.
31. Koya, S.F., et al., *Consumption of systemic antibiotics in India in 2019.* The Lancet Regional Health-Southeast Asia, 2022. **4**.
32. Sekoni, K.F., I.A. Oreagba, and F.A. Oladoja, *Antibiotic utilization study in a teaching hospital in Nigeria.* JAC-Antimicrobial Resistance, 2022. **4**(5): p. dlac093.
33. Islam, M.A., et al., *Pattern of antibiotic dispensing at pharmacies according to the WHO Access, Watch, Reserve (AWaRe) classification in Bangladesh.* Antibiotics, 2022. **11**(2): p. 247.
34. Aljadeeah, S., V.J. Wirtz, and E. Nagel, *Outpatient antibiotic dispensing for the population with government health insurance in Syria in 2018–2019.* Antibiotics, 2020. **9**(9): p. 570.
35. Saleem, Z., et al., *Sale of WHO AWaRe groups antibiotics without a prescription in Pakistan: a simulated client study.* Journal of pharmaceutical policy and practice, 2020. **13**: p. 1-8.
36. Nguyen, T.T.P., et al., *A national survey of dispensing practice and customer knowledge on antibiotic use in Vietnam and the implications.* Antibiotics, 2022. **11**(8): p. 1091.
37. Mehta, A., et al., *Systemic antibiotic sales and WHO recommendations, India.* Bulletin of the World Health Organization, 2022. **100**(10): p. 610.
38. Saleem, Z., et al., *WHO key access antibiotics prices, availability and affordability in private sector pharmacies in Pakistan.* Cost Effectiveness and Resource Allocation, 2021. **19**(1): p. 1-10.
39. Dat, V.Q., et al., *Purchase and use of antimicrobials in the hospital sector of Vietnam, a lower middle-income country with an emerging pharmaceuticals market.* PloS one, 2020. **15**(10): p. e0240830.
40. Pwint, K.H., et al., *Decreasing trends in antibiotic consumption in public hospitals from 2014 to 2017 following the decentralization of drug procurement in Myanmar.* Tropical Medicine and Infectious Disease, 2021. **6**(2): p. 57.
41. Amponsah, O.K.O., et al., *High levels of outpatient antibiotic prescription at a district hospital in Ghana: results of a cross sectional study.* International journal of environmental research and public health, 2022. **19**(16): p. 10286.
42. Priyadharsini, R., K. Ramasamy, and S. Amarendar, *Antibiotic-prescribing pattern in the outpatient departments using the WHO prescribing indicators and AWaRe assessment tool in a*

tertiary-care hospital in South India. Journal of Family Medicine and Primary Care, 2022. **11**(1): p. 74-78.
43. Rafi, S., et al., *Availability of Access, Watch, and Reserve groups of essential antibiotics: a cross-sectional survey.* Frontiers in Public Health, 2024. **11**: p. 1251434.
44. Mandal, P., et al., *Assessment of use of World Health Organization access, watch, reserve antibiotics and core prescribing indicators in pediatric outpatients in a tertiary care teaching hospital in Eastern India.* Perspectives in Clinical Research, 2023. **14**(2): p. 61.
45. Saleem, Z., et al., *Antibiotic consumption at community pharmacies: a multicenter repeated prevalence surveillance using WHO methodology.* Medicine access@ point of care, 2021. **5**: p. 23992026211064714.
46. Amaha, N.D., D.G. Weldemariam, and Y.H. Berhe, *Antibiotic consumption study in two hospitals in Asmara from 2014 to 2018 using WHO's defined daily dose (DDD) methodology.* PloS one, 2020. **15**(7): p. e0233275.
47. Knowles, R., et al., *Measuring antibiotic availability and use in 20 low-and middle-income countries.* Bulletin of the World Health Organization, 2020. **98**(3): p. 177.
48. Mudenda, S., et al., *Antibiotic prescribing patterns in adult patients according to the WHO AWaRe classification: a multi-facility cross-sectional study in primary healthcare hospitals in Lusaka, Zambia.* Pharmacology and Pharmacy, 2022. **13**(10): p. 379-392.
49. Negi, G., A. KB, and P.K. Panda, *Ground level utility of AWaRe Classification: Insights from a Tertiary Care Center In North India.* medRxiv, 2023: p. 2023.08. 02.23293536.
50. Mudenda, S., et al., *Prescribing patterns of antibiotics according to the WHO AWaRe classification during the COVID-19 pandemic at a teaching hospital in Lusaka, Zambia: implications for strengthening of antimicrobial stewardship programmes.* Pharmacoepidemiology, 2023. **2**(1): p. 42-53.
51. Boone, K., et al., *Antimicrobial Prescribing during Infant Hospital Admissions in a Birth Cohort in Dhaka, Bangladesh.* Journal of Tropical Pediatrics, 2021. **67**(3): p. fmaa093.
52. Sajjad, U., et al., *Evaluation of antibiotic prescription patterns using WHO AWaRe classification.* Policy and practice, 2024. **30**(2-2024).
53. Nguyen, N.V., et al., *Community-level consumption of antibiotics according to the AWaRe (Access, Watch, Reserve) classification in rural Vietnam.* JAC-antimicrobial resistance, 2020. **2**(3): p. dlaa048.
54. Okoro, R.N. and Z. Lawal, *Antibiotic utilization and resistance patterns in a secondary care hospital in Nigeria.* Saudi Journal of Clinical Pharmacy, 2023. **2**(3): p. 84-93.
55. Wieters, I., et al., *Reported antibiotic use among patients in the multicenter ANDEMIA infectious diseases surveillance study in sub-saharan Africa.* Antimicrobial Resistance & Infection Control, 2024. **13**(1): p. 9.
56. D'Arcy, N., et al., *Antibiotic prescribing patterns in Ghana, Uganda, Zambia and Tanzania hospitals: results from the global point prevalence survey (G-PPS) on antimicrobial use and stewardship interventions implemented.* Antibiotics, 2021. **10**(9): p. 1122.
57. Lakoh, S., et al., *Antibiotic use among hospitalized adult patients in a setting with limited laboratory infrastructure in Freetown Sierra Leone, 2017–2018.* International Journal of Infectious Diseases, 2020. **90**: p. 71-76.
58. Dereje, B., et al., *Prescribing Pattern and Associated Factors in Community Pharmacies: A Cross-Sectional Study Using AWaRe Classification and WHO Antibiotic Prescribing Indicators in Dire Dawa, Ethiopia.* Drugs-Real World Outcomes, 2023. **10**(3): p. 459-469.

59. Mustafa, Z.U., et al., *Knowledge, attitude and practices of self-medication including antibiotics among health care professionals during the COVID-19 pandemic in Pakistan: findings and implications.* Antibiotics, 2023. **12**(3): p. 481.
60. Melaku, T., et al., *Evaluation of adult outpatient antibiotics use at Jimma Medical Center (With defined daily doses for usage metrics).* Infection and Drug Resistance, 2021: p. 1649-1658.
61. Sri Ranganathan, S., et al., *A national survey of antibacterial consumption in Sri Lanka.* Plos one, 2021. **16**(9): p. e0257424.
62. Agyare, E., et al., *Antimicrobial stewardship capacity and antibiotic utilisation practices in the Cape Coast Teaching Hospital, Ghana: A point prevalence survey study.* Plos one, 2024. **19**(1): p. e0297626.
63. Khalfan, M.A., P.G. Sasi, and S.F. Mugusi, *The prevalence and pattern of antibiotic prescription among insured patients in Dar es Salaam Tanzania.* Pan African Medical Journal, 2021. **40**(1).
64. Nsojo, A., et al., *Prescribing patterns of antimicrobials according to the WHO AWaRe classification at a tertiary referral hospital in the southern highlands of Tanzania.* Infection Prevention in Practice, 2024. **6**(2): p. 100347.
65. Do, N.T., et al., *Community-based antibiotic access and use in six low-income and middle-income countries: a mixed-method approach.* The Lancet Global Health, 2021. **9**(5): p. e610-e619.
66. Valia, D., et al., *Use of WATCH antibiotics prior to presentation to the hospital in rural Burkina Faso.* Antimicrobial Resistance & Infection Control, 2022. **11**(1): p. 1-7.
67. Kanu, J.S., et al., *National antibiotic consumption for human use in Sierra Leone (2017–2019): a cross-sectional study.* Tropical medicine and infectious disease, 2021. **6**(2): p. 77.
68. SECHN, U.B., et al., *High levels of surgical antibiotic prophylaxis: Implications for hospital-based antibiotic stewardship in Sierra Leone.* 2022.
69. Labi, A.-K., et al., *Antibiotic consumption trends in Ghana: analysis of six-years pharmacy issue data from a secondary healthcare facility.* JAC-Antimicrobial Resistance, 2023. **5**(2): p. dlad025.
70. Murungi, M., et al., *Antimicrobial consumption surveillance in Uganda: Results from an analysis of national import data for the human health sector, 2018–2021.* Journal of Infection and Public Health, 2023. **16**: p. 45-51.
71. Lakoh, S., et al., *High levels of surgical antibiotic prophylaxis: implications for hospital-based antibiotic stewardship in Sierra Leone.* Antimicrobial Stewardship & Healthcare Epidemiology, 2022. **2**(1): p. e111.
72. Ngyedu, E.K., et al., *Selling antibiotics without prescriptions among community pharmacies and drug outlets: a simulated client study from Ghana.* Expert Review of Anti-infective Therapy, 2023. **21**(12): p. 1373-1382.
73. Khalid, M., J. Rasheed, and I. Nawaz, *Pattern of hospital antibiotic use in term neonates using WHO Access, Watch and Reserve Classification (AWaRe).* Pakistan Journal of Medical Sciences, 2022. **38**(8): p. 2169.
74. Nguyen, N.V., et al., *Outpatient antibiotic prescribing for acute respiratory infections in Vietnamese primary care settings by the WHO AWaRe (Access, Watch and Reserve) classification: An analysis using routinely collected electronic prescription data.* The Lancet Regional Health–Western Pacific, 2023. **30**.

75. Mambula, G., et al., *Practices and challenges related to antibiotic use in paediatric treatment in hospitals and health centres in Niger and Uganda: a mixed methods study.* Antimicrobial Resistance & Infection Control, 2023. **12**(1): p. 67.
76. Darkwah, T.O., et al., *Assessment of prescribing patterns of antibiotics using National Treatment Guidelines and World Health Organization prescribing indicators at the Ghana Police Hospital: a pilot study.* Pan African Medical Journal, 2021. **39**(1).
77. Tirfe, M., et al., *A three years antimicrobials consumption in Ethiopia from 2017 to 2019: A cross-sectional study.* Plos one, 2023. **18**(4): p. e0284038.
78. Abejew, A.A., G.Y. Wubetu, and T.G. Fenta, *A six years trend analysis of systemic antibiotic consumption in Northwest Ethiopia.* Plos one, 2024. **19**(1): p. e0290391.
79. Rockwood, N., et al., *A model for analysis of antibiotic usage in low-income settings.* Journal of Antimicrobial Chemotherapy, 2023. **78**(8): p. 2015-2018.
80. Mbwasi, R., et al., *National consumption of antimicrobials in Tanzania: 2017–2019.* Frontiers in Pharmacology, 2020. **11**: p. 585553.
81. Seni, J., et al., *Antimicrobial use across six referral hospitals in Tanzania: a point prevalence survey.* BMJ open, 2020. **10**(12): p. e042819.
82. Gautham, M., et al., *Availability, prices and affordability of antibiotics stocked by informal providers in rural India: a cross-sectional survey.* Antibiotics, 2022. **11**(4): p. 523.
83. Darwish Elhajji, F., et al., *Overview of Availability, Cost, and Affordability of Antibiotics for Adults in Jordan: An AWaRe Classification Perspective.* Antibiotics, 2023. **12**(11): p. 1576.
84. Zirpe, K.G., et al., *Impact of an antimicrobial stewardship program on broad spectrum antibiotics consumption in the intensive care setting.* Indian Journal of Critical Care Medicine: Peer-reviewed, Official Publication of Indian Society of Critical Care Medicine, 2023. **27**(10): p. 737.
85. Malik, F. and A. Figueras, *Continuous rise in cephalosporin and fluoroquinolone consumption in Pakistan: a 5 year analysis (2014–18).* JAC-Antimicrobial Resistance, 2019. **1**(3): p. dlz063.
86. Nsojo, A., et al., *Prescribing patterns of antimicrobials according to the WHO AWaRe classification at a tertiary referral hospital in the southern highlands of Tanzania.* Infection Prevention in Practice, 2024: p. 100347.
87. Mandal, P., et al., *Assessment of use of World Health Organization access, watch, reserve antibiotics and core prescribing indicators in pediatric outpatients in a tertiary care teaching hospital in Eastern India.* Perspectives in Clinical Research, 2023. **14**(2): p. 61-67.
88. Murungi, M., et al., *Antimicrobial consumption surveillance in Uganda: Results from an analysis of national import data for the human health sector, 2018–2021.* Journal of Infection and Public Health, 2023. **16**: p. 45-51.
89. Valia, D., et al., *Use of WATCH antibiotics prior to presentation to the hospital in rural Burkina Faso.* Antimicrobial Resistance & Infection Control, 2022. **11**(1): p. 59.
90. Mbwasi, R., et al., *National consumption of antimicrobials in Tanzania: 2017–2019.* Frontiers in Pharmacology, 2020. **11**: p. 585553.
91. Sajjad, U., et al., *Evaluation of antibiotic prescription patterns using WHO AWaRe classification.* Eastern Mediterranean Health Journal, 2024. **30**(2).
92. Bansal, A., R. Sharma, and R. Prakash, *Adoption of the World Health Organization access, watch reserve index to evaluate and monitor the use of antibiotics at a tertiary care hospital in India.* Perspectives in Clinical Research, 2022. **13**(2): p. 90-93.

93. Hussein, R.R., et al., *Antibiotic consumption in hospitals during COVID-19 pandemic: a comparative study.* The Journal of Infection in Developing Countries, 2022. **16**(11): p. 1679-1686.
94. Saleem, Z., et al., *WHO key access antibiotics prices, availability and affordability in private sector pharmacies in Pakistan.* Cost Effectiveness and Resource Allocation, 2021. **19**: p. 1-10.
95. Portal, W.A., *WHO AWaRe Antibiotic Categorization.* 2020.
96. Chansamouth, V., et al., *Implementing the WHO AWaRe antibiotic book guidance in lower-resource settings: the case of the Lao PDR.* JAC-Antimicrobial Resistance, 2024. **6**(1): p. dlae004.
97. Sharland, M., et al., *Classifying antibiotics in the WHO Essential Medicines List for optimal use—be AWaRe.* The Lancet Infectious Diseases, 2018. **18**(1): p. 18-20.
98. Chansamouth, V., et al., *Evaluation of trends in hospital antimicrobial use in the Lao PDR using repeated point-prevalence surveys-evidence to improve treatment guideline use.* The Lancet Regional Health - Western Pacific, 2022. **27**: p. 100531.
99. Sharland, M., et al., *The WHO essential medicines list AWaRe book: From a list to a quality improvement system.* Clinical Microbiology and Infection, 2022. **28**(12): p. 1533-1535.
100. Sharland, M., et al., *The WHO AWaRe Antibiotic Book: providing guidance on optimal use and informing policy.* The Lancet Infectious Diseases, 2022. **22**(11): p. 1528-1530.
101. Zanichelli, V., et al., *The WHO AWaRe (Access, Watch, Reserve) antibiotic book and prevention of antimicrobial resistance.* 2023.
